# I Think I Age Gracefully: A Focus Group Study of Successful Aging Conceptions Among Older Women Living With HIV

**DOI:** 10.1093/geroni/igab046.828

**Published:** 2021-12-17

**Authors:** Anna Rubtsova, Tonya Taylor, Gina Wingood, Igho Ofotokun, Deborah Gustafson, Marcia Holstad

**Affiliations:** 1 Emory University Rollins School of Public Health, Emory University, Georgia, United States; 2 SUNY Downstate Health Sciences University, Brooklyn, New York, United States; 3 Columbia University Mailman School of Public Health, New York, New York, United States; 4 Emory University School of Medicine, Atlanta, Georgia, United States; 5 Emory University, Atlanta, Georgia, United States

## Abstract

Our previous quantitative research found high prevalence of self-rated successful aging (SA) among older (age ≥50) women living with HIV (OWLH) enrolled in the Women’s Interagency HIV Study (WIHS). However, little is known about how OWLH define SA. Most studies have examined SA among predominantly white men living with HIV. Therefore, the purpose of our qualitative study was to examine subjective understandings of SA among OWLH and, as a comparison group, older HIV-seronegative women at risk of HIV. Four focus group discussions (FGD) were conducted among 23 participants (12 OWLH, 11 HIV-seronegative). These women were recruited from WIHS participants previously enrolled in our quantitative study of SA, “From Surviving to Thriving” (FROST), at two WIHS sites – Atlanta and Brooklyn. At each site, we conducted two FGD – one with OWLH and one with older HIV-seronegative women in February-March of 2019. Participants were, on average, 56 years old (range, 51-70), 78% Black, and 60% with annual income ≤ $12,000. A team of coders conducted thematic coding of fully transcribed FGD using MAXQDA software. Several themes emerged. Both OWLH and older HIV-seronegative women defined SA as “aging gracefully,” i.e. accepting and celebrating aging after having survived hardships of earlier life (e.g., HIV diagnosis, drug use). They also emphasized taking care of themselves (e.g., taking their meds) and spirituality in their definitions of SA. In contrast to HIV-seronegative participants, who prioritized sobriety as taking good care of themselves, OWLH emphasized taking care of their HIV (e.g., “staying on top of your numbers”).

